# Utilizing of KNO_3_, KCl, and NaCl as phase change materials within the refrigerator for energy saving

**DOI:** 10.1038/s41598-026-41662-5

**Published:** 2026-03-24

**Authors:** Sami Samir, Mahmoud Salem, A. S. A. Mohamed, Ahmed Askalany

**Affiliations:** Mechanical Department, Faculty of Technology and Education, Sohag University, Sohag, 82524 Egypt

**Keywords:** PCM, COP, Home refrigerator, Evaporator, Condenser

## Abstract

Practical experiments were conducted on a home refrigerator with a capacity of 289 L, under hot weather conditions, to improve energy consumption and coefficient of Performance (COP) using several phase change materials (PCMs). A comparison was made between different materials (KNO_3_, KCl, and NaCl) at a mixture ratio of 10% with 90% H_2_O, and using H_2_O. A stainless-steel container with volumes of 1 L and 1.5 L was placed under the evaporator, without sacrificing any space for freezing or cooling. PCM materials have demonstrated their effectiveness in minimizing temperature variations within a refrigeration environment. The results indicate that using a 1 L KNO_3_ mixture achieved the most significant improvement, increasing COP by 12.1% and reducing electricity consumption by 8.6%. The KCl mixture came in second place, followed by NaCl, while the least effective was H_2_O. All results showed that PCM contributes to reducing compressor operating periods by up to 7.1%.

## Introduction

The operation of cooling and air conditioning systems, along with lighting, accounts for approximately 40% of worldwide energy use^1^. Egypt is facing significant challenges due to increased energy demand resulting from population growth, industrial expansion, and the higher use of electrical appliances, especially during the summer. Has led to frequent power outages. Among the most demanding appliances, refrigerators operate continuously throughout the year. As a result, refrigerators continue to provide significant energy-saving potential^2^. Improved cabinet and door thermal insulation^3–6^, and improved condenser and evaporator heat transfer^7–9^. Several technological advancements have been made to enhance the performance of residential refrigerators. The use of PCMs is promising and requires further research to enhance their effectiveness in energy conservation^10,11^. They are materials with efficient thermal storage^12^. Reduces compressor operating times^13^. Energy consumption is reduced, and the refrigerator’s (COP) is enhanced when PCM is applied to the evaporator^7,14^. It is an effective solution to combat climate change^15^. The implementation of PCM in the condenser of the refrigerator resulted in a 12% enhancement in energy efficiency^16^. It also contributes to promoting the goals of sustainable development^17^. Adding phase change materials (PCMs) to the components of refrigerator compartments is an effective way to improve system performance and minimize temperature variations between sections^18–20^.

Sonnenrein et al.^21^ studied the impact of three types of PCM (paraffin, water, and copolymer compound) on a refrigerator condenser. The results indicated a reduction in condenser temperature, an improvement in COP, and a 10% decrease in energy consumption.

Yusufoglu et al.^22^ tested four phase change material (PCM) varieties in two residential refrigerator models, one with a 130 L capacity and the other with 350 L, both using R600a refrigerant. The first model had 1.8 kg of PCM in a plate-shaped container, while the second had 0.950 kg. Results showed a 2–4 °C increase in temperature in both the evaporator and condenser for all PCM types, with compressor efficiency improving by 8.8% in the first model and 9.4% in the second.

A computational model using PCM N-Octadecane at 27.5 °C, after the condenser and before the expansion valve, was conducted by Valipour et al.^20^, and the findings showed a 9.58% improvement in COP.

Elarem et al.^23^ experimented with a U-shaped copper tube heat exchanger beneath the evaporator, using A4 PluseICE phase change material. This setup improved the COP by 8% and reduced energy consumption by 12%. Additionally, CFD simulations of the PCM’s placement in the refrigerator showed an 86.66% improvement in performance.

Berdja et al.^24^ investigated a 400 L refrigerator featuring a 10 mm thick PCM at -10 °C on the evaporator surface. Their findings showed improved heat transfer efficiency, increased coefficient of performance, and reduced electrical consumption.

Vasco et al.^13^ assessed the effect of PCMs on the efficiency of the household refrigerator evaporator. They used a commercial mixture, E-10, and a composition of 19.5% NH_4_Cl and 80.5% H_2_O in 12 parallel tubes. Results showed a 5.81% reduction in energy consumption with 19.5% NH_4_Cl, while E-10 led to a 1.74% decrease.

Pirvaram et al.^25^ investigated phase change materials (PCMs) in refrigerator condensers, utilizing polyethylene glycol with molecular weights of 600 and 1000, with a thermal load of 10 kg. Their results showed improved condenser performance, with a temperature drop from 37.5 to 34.5 °C and a 13% reduction in energy consumption compared to traditional systems.

Sharma et al.^26^ used ANSYS simulations to study the effects of PCMs in domestic refrigerators, placing them at the base of each tray. Their findings showed that PCMs improved temperature stability by reducing fluctuations and extending compressor shut-off times. The most effective PCM configurations, both vertical and horizontal, covered over 10% of the system’s total area.

Kasinathan et al.^27^ compared the utilization of PCMs and their effect on energy when using OM46 paraffin in the condenser and natural graphite (3 wt.%) in an evaporator against a standard refrigerator. They found reductions of 7% to 13% in the evaporator and 16% to 22% in the condenser, indicating that PCM, especially in the condenser, offers significant energy savings.

Sekhar et al.^28^ tested seven configurations of PCMs on the evaporator and condenser. The maximum optimization achieved an 18.5% reduction in energy consumption.

Ben Taher et al.^29^ Investigated eutectic solutions and water as PCMs and the effect of PCM thickness on energy efficiency. They found that a 7 mm-thick PCM allows 720 min of compressor downtime with water, while maintaining temperatures between 4 and 6 °C for both the water and the eutectic solution.

Mane et al.^30^ Conducted experiments with different masses of NaCl, KCl, and NaF solutions dissolved in water inside and below the evaporator during door opening and closing. The results indicated an increase in the equivalent energy consumption due to door opening and a decrease with increasing PCM masses.

Rashid et al.^31^ integrated PCM in the refrigerator’s condenser and evaporator, resulting in a 12.7% increase in COP, a 25.1% reduction in energy consumption, and a 1.5-fold increase in compressor off time.

Nigussie et al.^32^ Performed experiments involving three distinct types of PCM (CaCl2⋅6H_2_O, NaCl, and paraffin wax). Between the condenser and the evaporator, or both. The results indicated that COP increased by 9% with NaCl in the evaporator, 34% with a 2 mm thickness of paraffin wax in the condenser, and 27% when PCM was combined on both the condenser and the evaporator.

Shafii et al.^33^ presented a 24-h dynamic model for the combination of a PCM reservoir (eutectic mixtures, CaCl2.6H_2_O, oleic acid, and a commercial blend).in a(VCR) system. The stored energy was reused for condenser inlet cooling, reducing electricity consumption during peak periods. The results showed a 23.38% reduction in electricity consumption.

The significance of employing phase change materials in refrigeration systems has been emphasized by earlier research. Nevertheless, their study did not employ conventional experimental settings and focused on three specific materials (NaCl, KCl, and H_2_O). This study aims to use KNO_3_ as a PCM to improve the performance of household refrigerators under realistic operating conditions. It also presents a comparison between KNO_3_ and (NaCl, KCl, and H_2_O). The PCM KNO3 has not been thoroughly studied in previous research; however, it falls within the appropriate temperature range for analysis. Using two volumes (1 L and 1.5 L), A thorough examination of these materials’ performance is provided, along with information on how they affect COP, energy usage, internal temperatures, and the surface temperatures of the compressor and Condenser.

## Experimental work

**Fig. 1:**
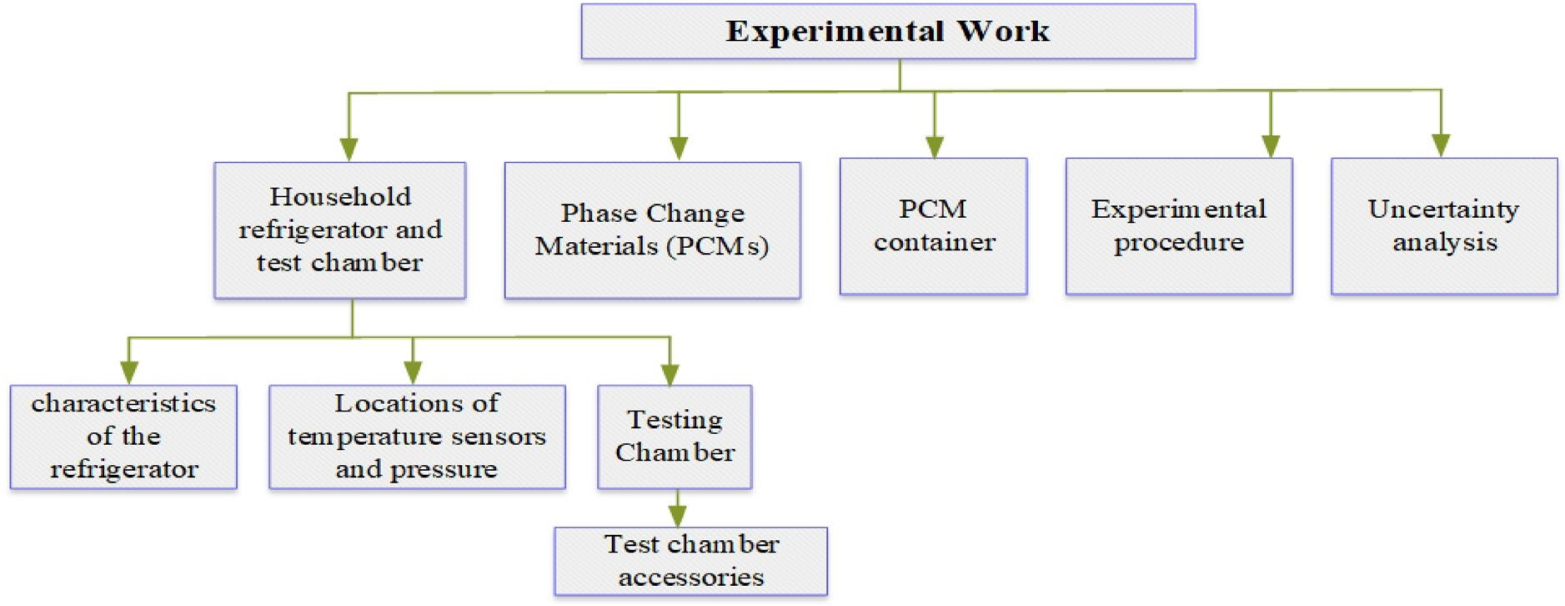
Schematic of experimental work.

This section provides a detailed overview of the experimental methods used in the research, as illustrated in Fig. 1.

### Household refrigerator and test chamber

#### Characteristics of the refrigerator

**Fig. 2:**
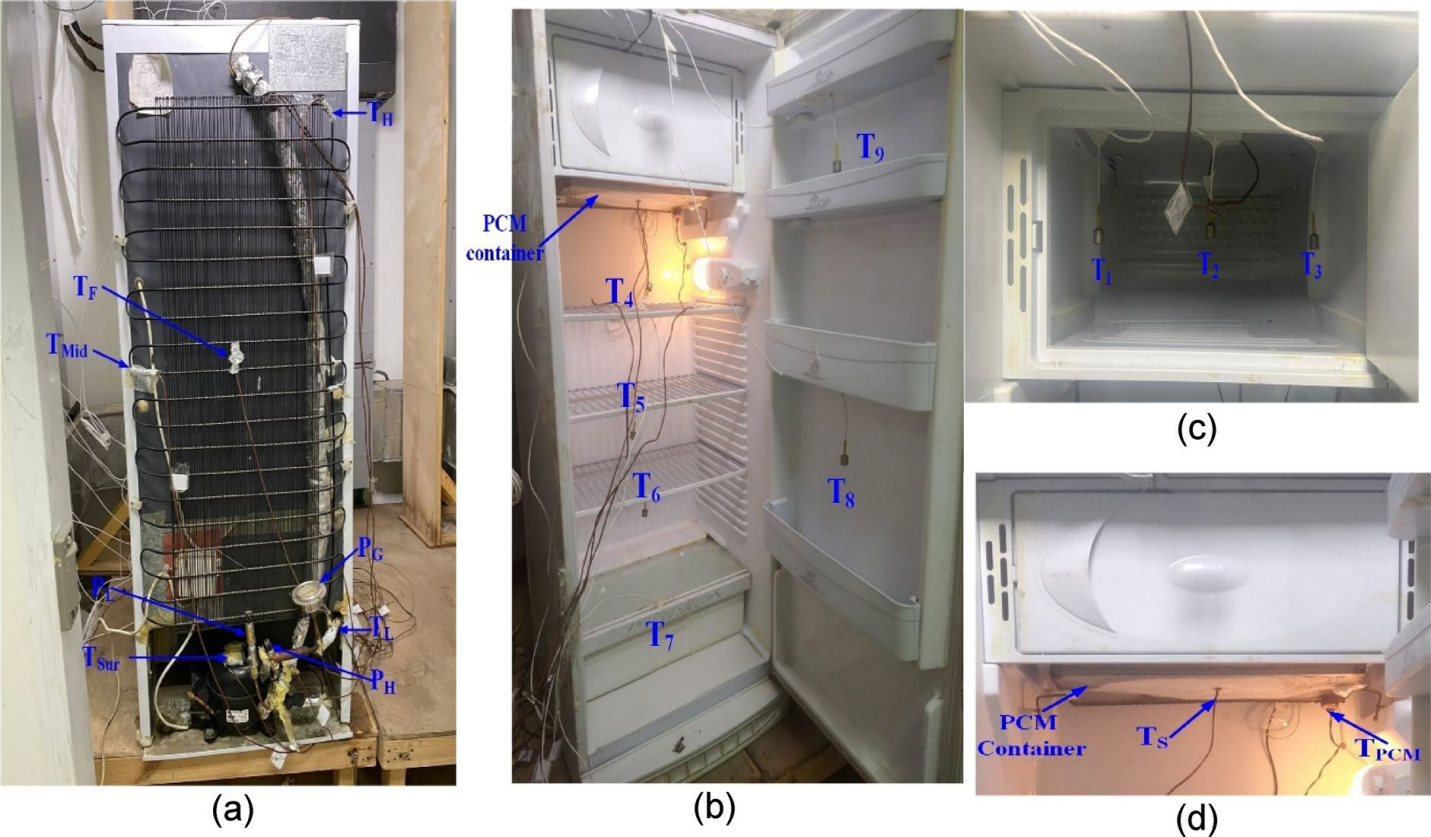
Locations of temperature sensors and pressure measurements throughout the refrigerator: (**a**) compressor and condenser, (**b**) compartment and door, (**c**) freezer, (**d**) PCM container.

A single-door refrigerator with a capacity of 289 L was utilized. It includes a cooling and freezing compartment, a cooling compartment that divides the refrigerator into three equal shelves, and a fresh vegetable compartment (see Fig. 2). Table 1 lists the refrigerator’s specifications.

**Table 1:** The characteristics of the refrigerator.

Dimensions (mm)	1450 × 600 × 560
Evaporator:	Free convection
Condenser	Free convection
Compressor	ZMC-EGM77AF-220-204 V-50 Hz
Expansion device	Capillary tube
Refrigerant	R-134a

#### Locations of temperature sensors and pressure

**Fig. 3:**
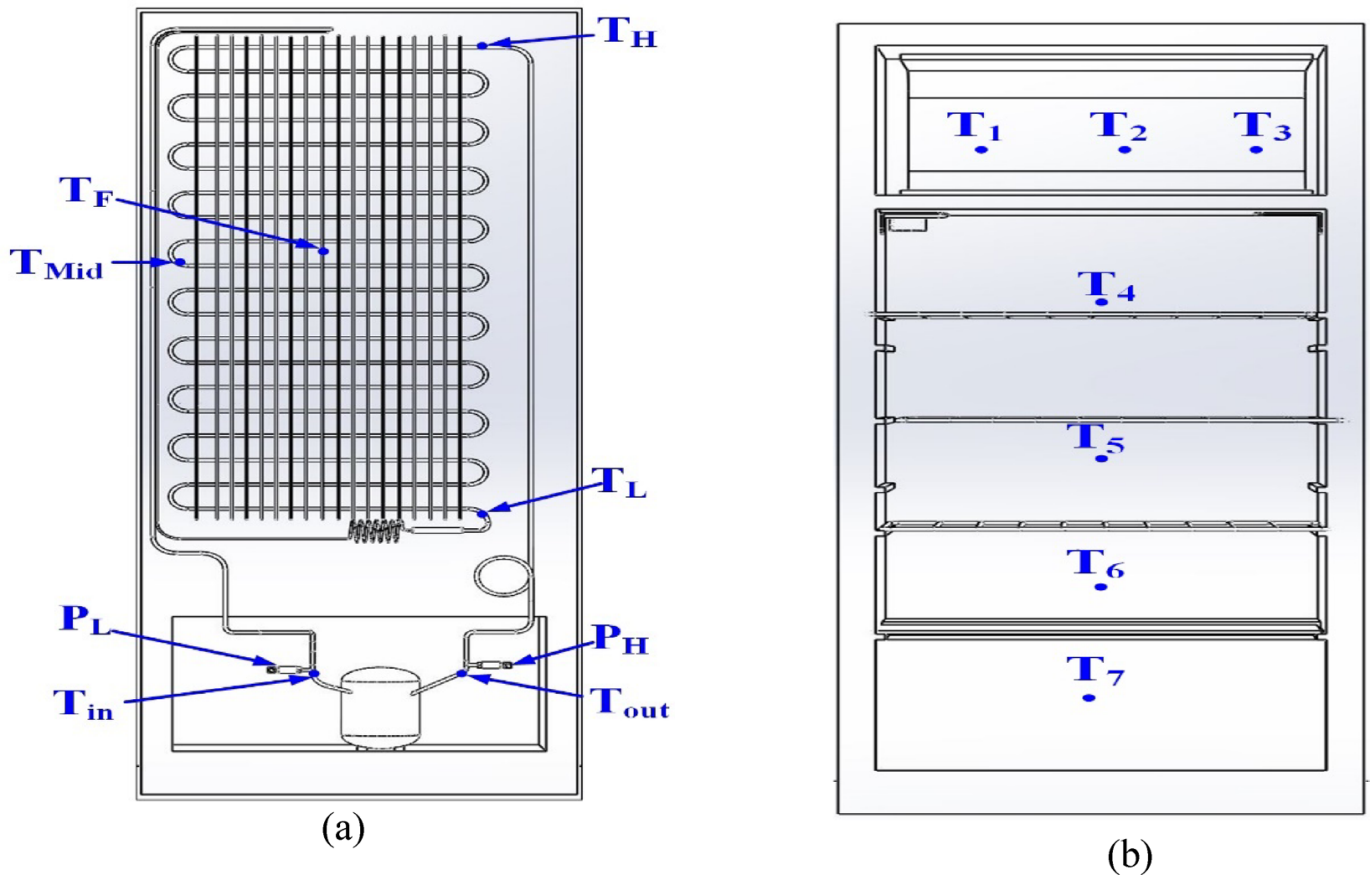
Schematic locations of temperature sensors and pressure measurements throughout the refrigerator: (**a**) compressor and condenser, (**b**) compartment.

Temperature and pressure measurements are taken at various points in the refrigeration system, including transducers at the compressor’s inlet and outlet and temperature checks at the condenser, compressor surface, compartments, and freezer (see Fig. 2a–c). Several Class A +  + PT100 temperature sensors are strategically positioned in the compartments, door, freezer, and use-temperature sensor type T in the PCM container (see Fig. 3) to assess performance both with and without PCM. Uncertainty ratio in temperature and pressure: ± 0.5 °C and ± 0.01 bar, respectively. A schematic of the positions of the various temperature and pressure readings on the refrigerator is provided in Fig. 3.

#### Testing chamber

**Fig. 4:**
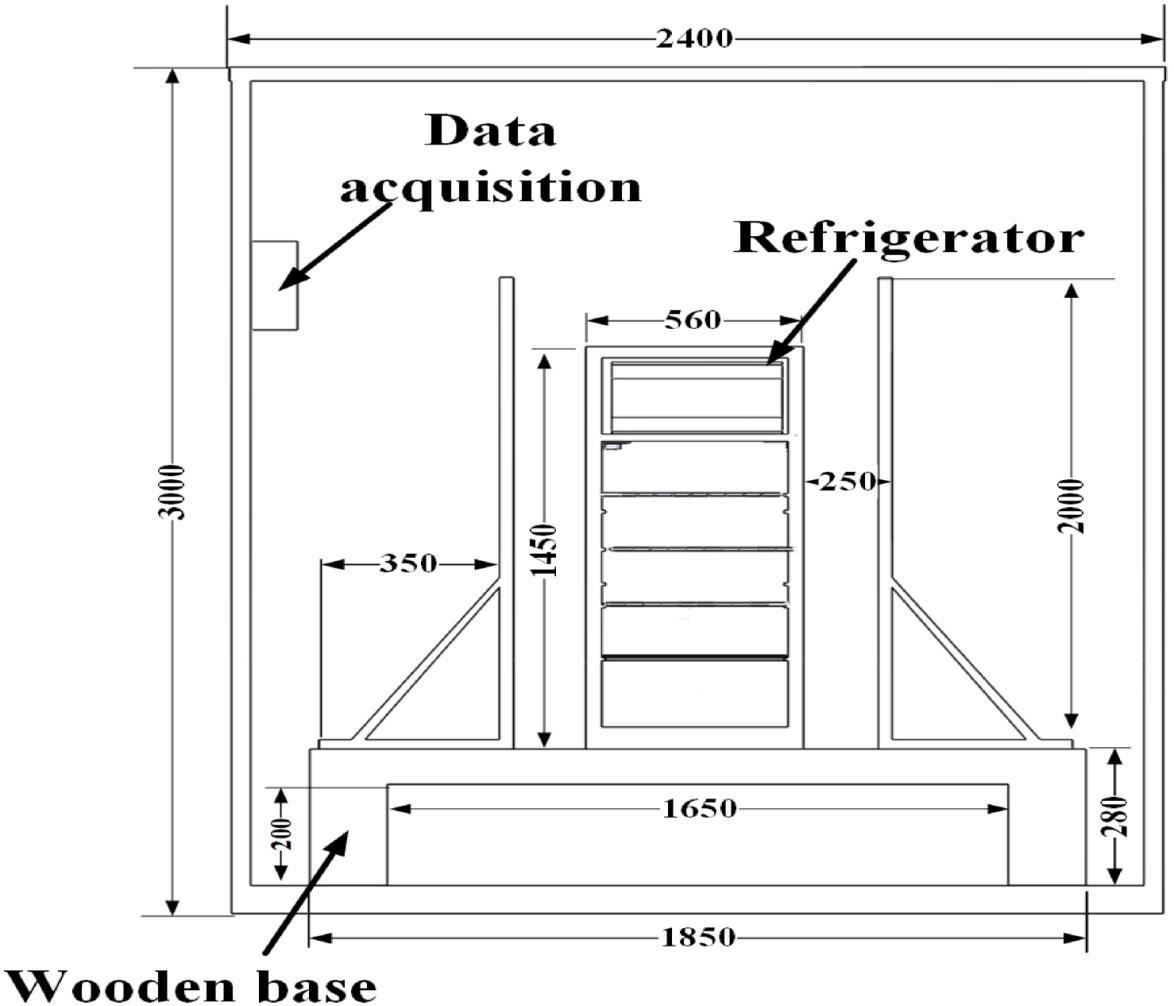
Position the test refrigerator within the test chamber.

The research experiments were conducted in the Energy Efficiency Measurement Laboratory’s Refrigeration and Air Conditioning Laboratory, which holds an International Certificate from the Egyptian General Accreditation Council (EGAC) and is affiliated with the Faculty of Technology and Education, Sohag University, Egypt. The International Organization for Standardization’s ISO 17,027:2017/IEC 62,552 experimental conditions are followed in this process. The temperature in the test room was maintained at 32 °C and the humidity of 55%. It has dimensions of 3000 mm × 2400 mm × 2000 mm and is thermally insulated with fiberglass, equipped with a tightly closed door (Fig. 4) to maintain an airflow rate of 0.25 m/s. The chamber construction features Foam-core panel walls, which offer several advantages over traditional systems. These panels provide excellent insulation, resist moisture without significantly affecting their R-value, and minimize air convection and thermal bridging.

The test refrigerator is mounted on a wooden base within the test chamber, elevated to mitigate the chamber’s ground effect. It is arranged according to the relative dimensions specified in the IEC 62,552 standard (Fig. 4).

##### Test chamber accessories

Contain the refrigerator test unit:

- **Air Handling Unit:** for adjusting the chamber temperature and humidity according to test settings by the IEC Standard.
- **Power Analyzer**: to read and display actual power data from feeding samples while testing.
- **Data Acquisition**: to measure 16 RTD Temperature Sensors.
- **AC Power Supply:** individual Variable Power Supply for Each Unit under test.
- **Temperature Sensors:** Tin-plated copper with precision machining to meet the IEC62552 criteria, Sensor type PT100, class A +  + .

### Phase change materials (PCM)

**Fig. 5:**
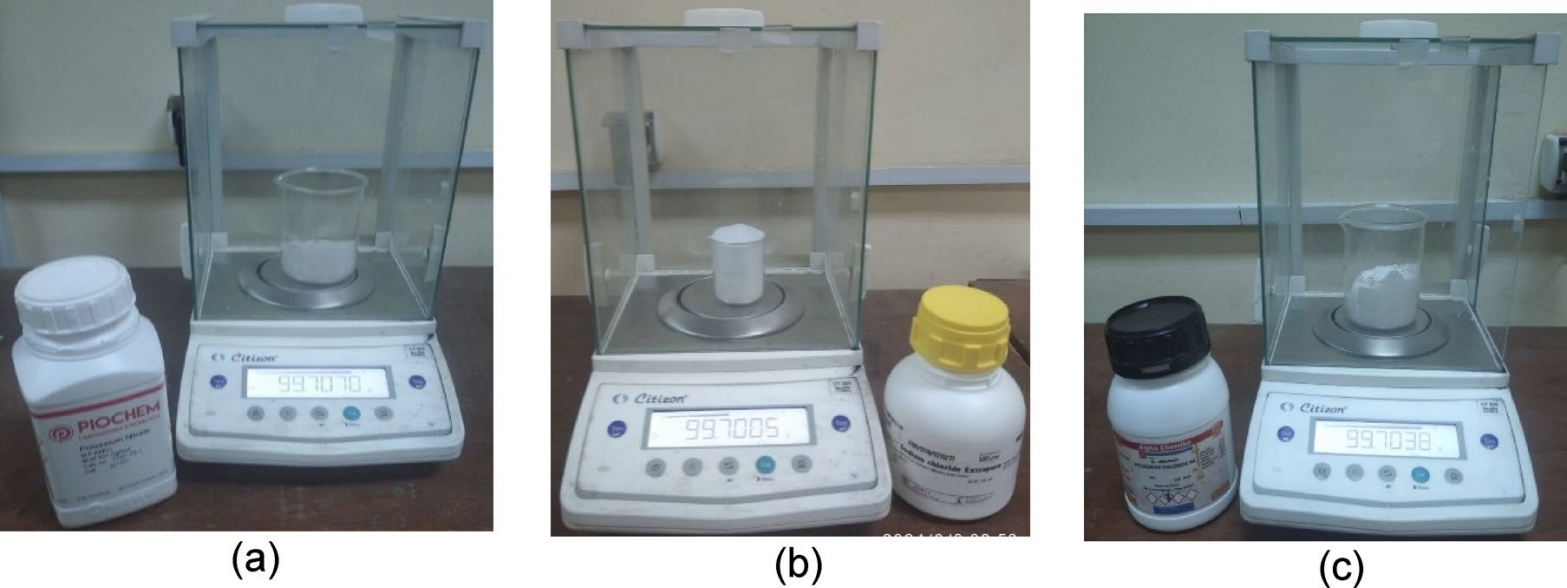
Weight of phase change material in 1 L experiments. (**a**) KNO_3_, (**b**) NaCl, (**c**) KCl.

Phase Change Materials (PCMs) are capable of storing and releasing substantial latent thermal energy during their phase transitions^34^. This characteristic renders them highly beneficial for applications in thermal energy storage, product temperature regulation, and refrigeration systems^35,36^. A preliminary test was conducted on the refrigerator under study to determine its temperature range and then search for the concentration of solutions in this range. Four different PCMs (10% NaCl + 90% H_2_O) wt.., (10% KCl + 90% H_2_O) wt.., (10% KNO_3_ + 90% H_2_O) wt.., and pure 100% H_2_O are used under the evaporator of a refrigerator. The density of distilled water at 25 °C was determined using standard water property tables, yielding a value of 996.99 kg/m^3^. Based on this, 0.997 kg of distilled water was measured, corresponding to 1 L. (Fig. 5). Weight of 10% PCM in 1 L experiments. The thermophysical properties (melting point and latent heat) are not experimentally validated in this study; these values are derived from the literature. The Physical characteristics of PCMs are given in Table 2.

**Table 2:** Physical characteristics of PCM^30,37–39^.

PCMs	Concentration (%wt.)	Melting point temperature (°C)	Latent heat of fusion (kJ/kg)	Cost ($\kg)
Pure H_2_O	100% H_2_O	0	333	–
Eutectic 1	(90% H_2_O + 10% KNO_3_)	− 4.1	333.6	19.95
Eutectic 2	(90% H_2_O + 10% NaCl)	− 5.0	289	6.92
Eutectic 3	(90% H_2_O + 10% kCl)	− 6.6	283	17.79

### PCM container

**Fig. 6:**
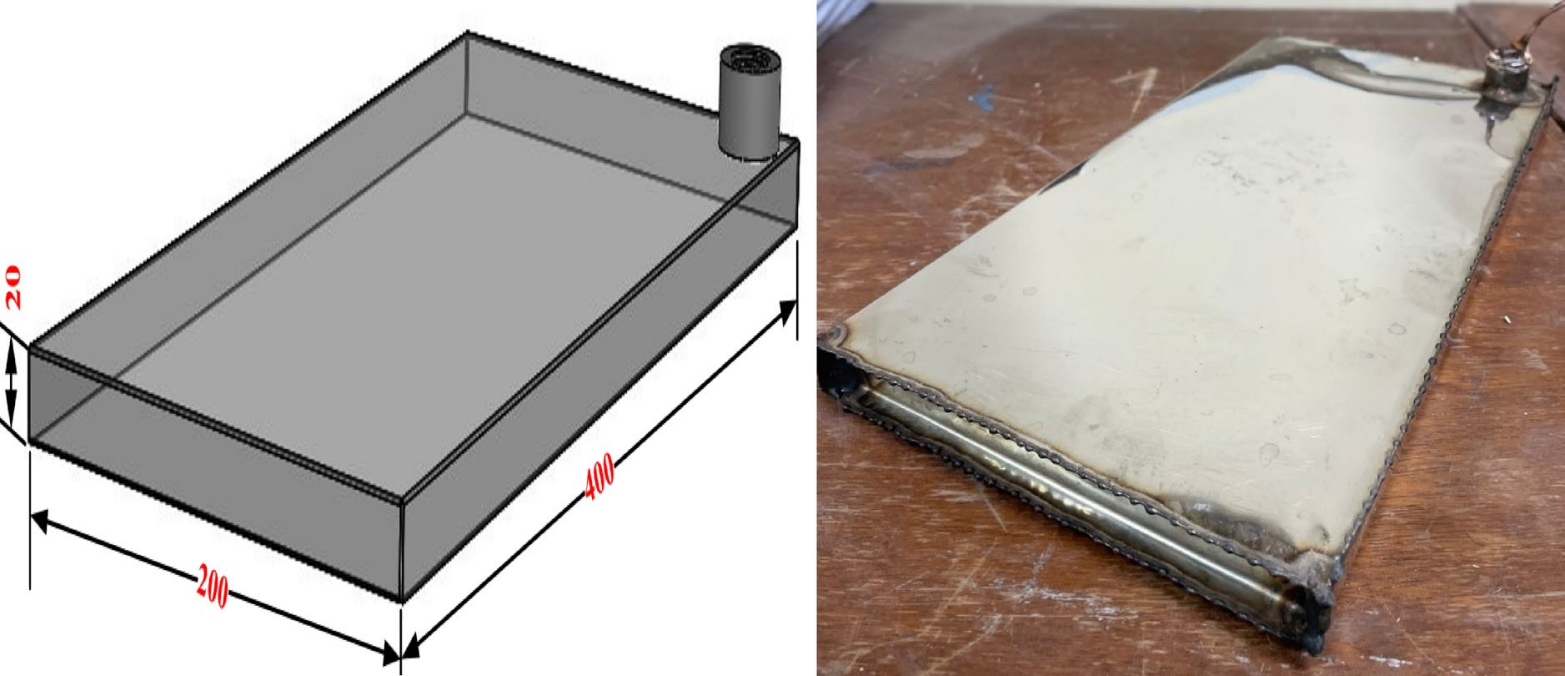
PCM container.

A container made of 304 stainless steel plates with a thickness of 0.4 mm and dimensions of 400 × 200 × 20 mm was used (Fig. 6). These solutions and their proportions are stored in stainless steel without undergoing oxidation. The container was sealed tightly to prevent any possible leakage, thereby ensuring both experimental safety and stability. Its thermal conductivity measures 16.2 W/m.K. It was sized to fit between the refrigerator’s cooling section and the freezer, without sacrificing any space for freezing or cooling.

### Experimental procedure

**Fig. 7:**
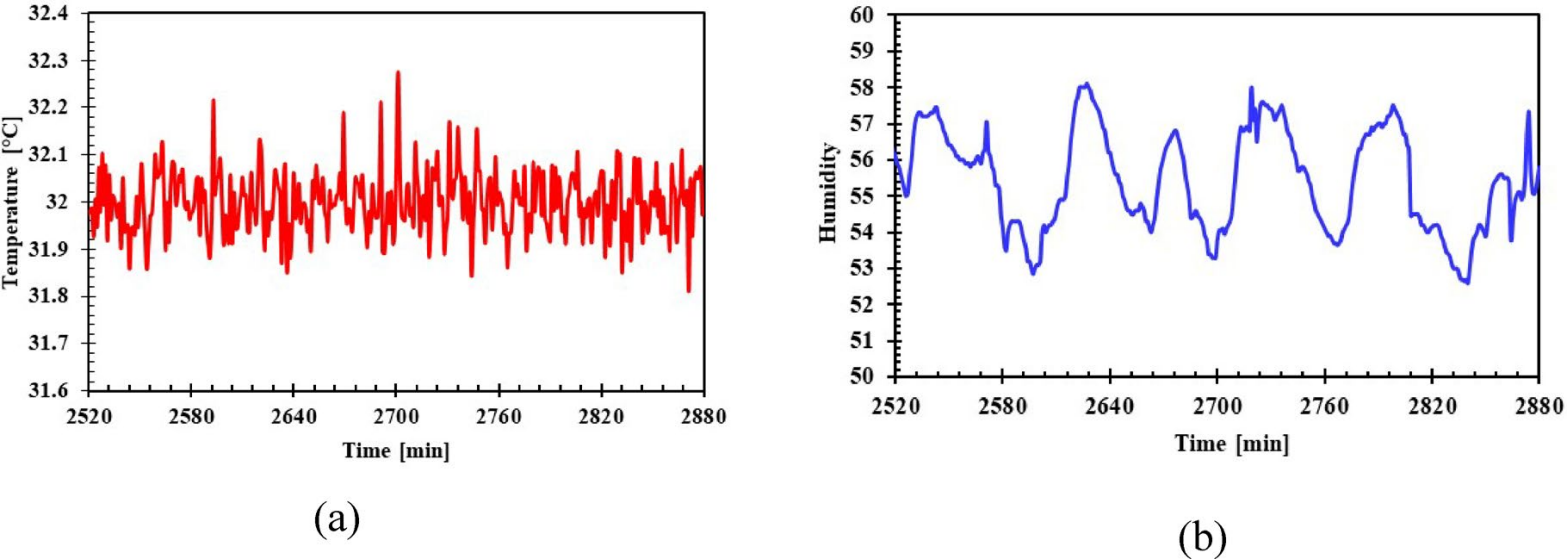
Ambient conditions (**a**) Temperature, (**b**) Humidity.

The eight cases, both with and without PCM (Table 3), will be tested to assess the refrigerator’s performance under identical operating conditions (Table 4). The tests were run three times for each test, allowing them to calculate the mean value from their recorded measurements. The experiments were conducted in a controlled environment room, where both the temperature and humidity were consistently regulated at 32 °C and 55%, respectively (Fig. 7) The air conditioning unit in the test room was operated for 120 min prior to the start of the test to ensure that the required temperature and humidity were achieved. It continues to operate during the test period to maintain the test conditions. The refrigerator is operated for 2880 min, while maintaining testing conditions, with the refrigerator’s internal temperatures, as well as the condensation and evaporation pressures, Compressor, condenser, and evaporator temperatures, being recorded. The power supply and energy consumption of the refrigerator were measured using data acquisition systems. The compressor’s surface temperature and the condenser mid temperature. They were also measured to examine the operational parameters in more detail. All this data collection is based on the refrigerator being in a steady-state condition (After 24 h from the start of operation, we observed that the thermal stability maintained a steady temperature during on/off cycles; the temperature changes were within ± 0.2 °C). The system adhered to its on/off times. Then, the power is cut off while the internal temperature of the refrigerator is continued to be recorded for 60 min. To evaluate the impact of PCM integration on the refrigerator’s internal temperatures. While maintaining the testing conditions as specified in Table 4.

**Table 3:** List of experiments.

Case	PCM	Concentration, wt	Volume, L
0	Without PCM	–	–
1	Water	100% (H_2_O)	1
2	Eutectic solution1	10% KNO_3_ + 90% H_2_O	1
3	Eutectic solution 2	10% NaCl + 90% H_2_O	1
4	Eutectic solution 3	10% KCl + 90% H_2_O	1
5	Water	100% (H_2_O)	1.5
6	Eutectic solution1	10% KNO_3_ + 90% H_2_O	1.5
7	Eutectic solution 2	10% NaCl + 90% H_2_O	1.5
8	Eutectic solution 3	10% KCl + 90% H_2_O	1.5

**Table 4:** Test conditions.

T_amb_ (°C)	32 (± 0.5)
Rh %	55 (± 5)
Power supply (V)	220
Frequency (Hz)	50
Test time (min)	2880

### Uncertainty analysis

To address measurement errors in the experiment. The uncertainty is evaluated by calculating the cumulative uncertainty (Uc), which encompasses the uncertainties of repeatability (Urep), calibration (Ucal), drift (Ud), and resolution (Ures). According to the following equation:

$$Uc=\sqrt{{\left(Urep\right)}^{2}+{\left(Ucal\right)}^{2}+{\left(Ud\right)}^{2}}+{\left(Ures\right)}^{2}$$

The estimated UC was 1.4%. The Uc is multiplied by a coverage factor (k) of 2 to obtain an expanded uncertainty, corresponding to a confidence level of approximately 95% (95.45%). Table 5 shows the accuracy of the devices used in the experiment.

**Table 5:** Accuracy of the devices used in the experiment.

Devices	Accuracy
Thermocouple type T	± 0.5 °C
Room temperature and humidity sensor AF5485	± 0.4 °C, ± 1.7%
Data logger	± 0.1%
Pressure transmitter	± 0.125%

## Results and discussion

This section analyzes experimental results, detailing temperature measurements from various refrigerator locations, including the condenser, compressor surface, freezer interior, compartments, vegetable drawer, and door, as shown in Fig. 2. Temperature recordings are conducted both with and without PCMs for the various cases mentioned earlier. The impact of PCM on refrigerator temperatures during a power outage has also been tested. The effect of PCM on compressor operation is studied. The analysis compares the refrigerator’s performance with and without PCMs.

### The impact of PCMs on temperature distribution in the refrigerator

#### Mid-condenser temperature

**Fig. 8:**
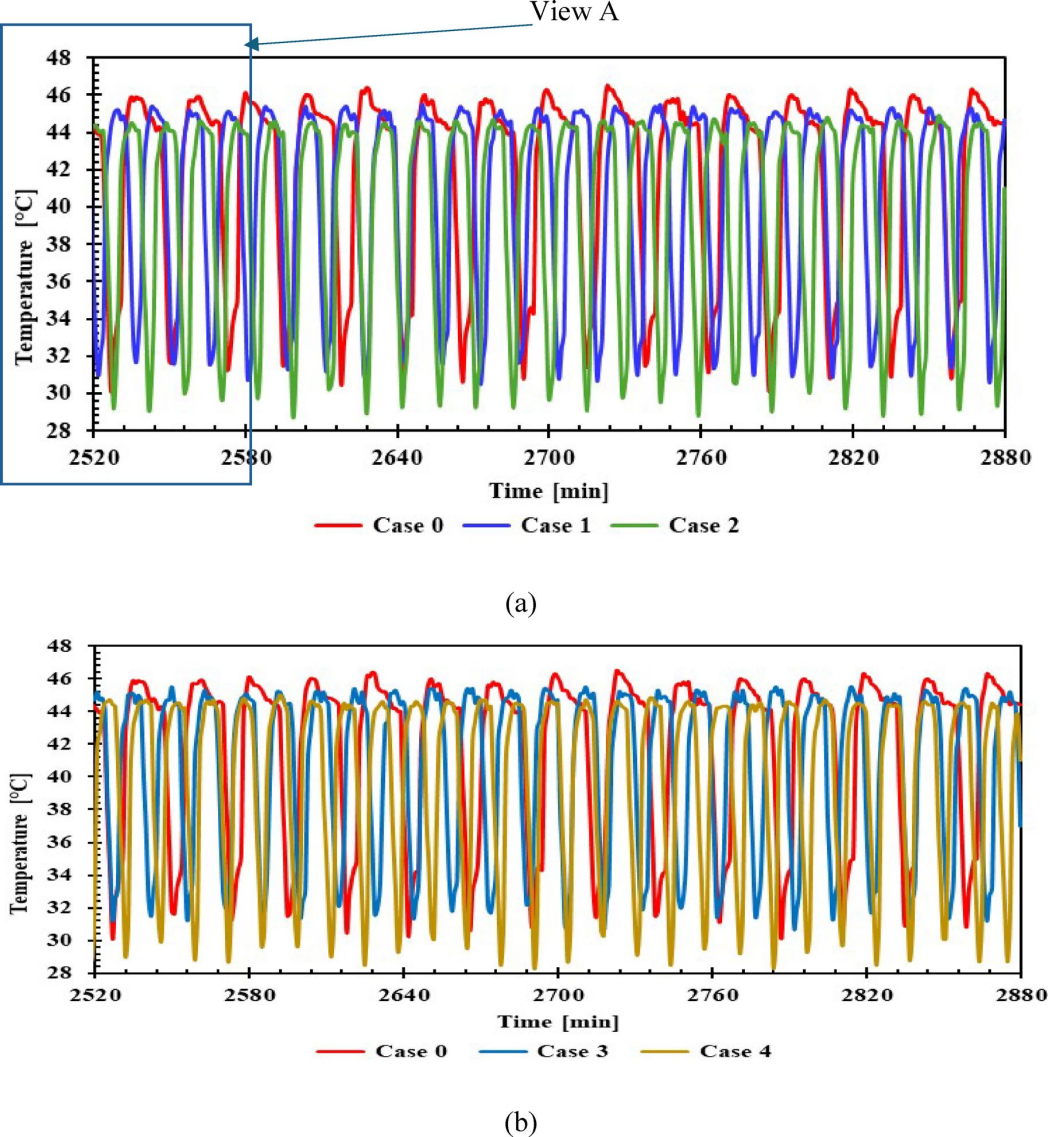

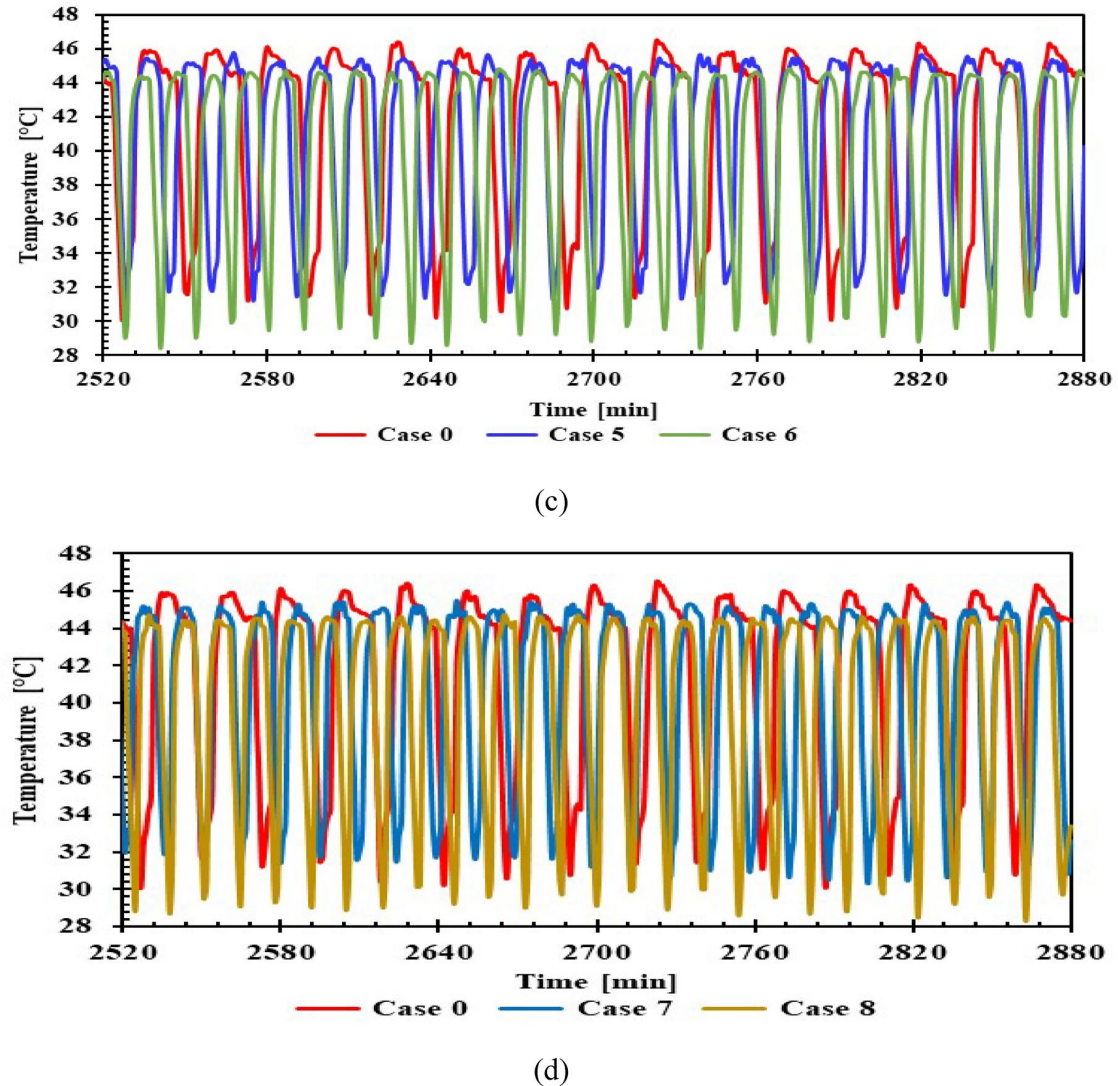
Temperature of condenser midpoint with and without PCMs on/off cycle over Time (a, b) 1 L, (c, d) 1.5 L.

The comparison of condenser midpoint temperatures for different cases is shown in Fig. 8, with 1 L and 1.5 L PCM and without PCM. In case 0, the condenser midpoint temperature during compressor operation reached its highest value (about 46.2 °C) before the compressor stopped. For the PCM refrigerator, the condenser midpoint temperature decreased by 1.6 °C compared to case 0. While in the off state, the temperature reached 31.6 °C in case 0. Using PCM, the temperature decreased by 3 °C and 2 °C with volumes of 1 L and 1.5 L, respectively. Resulted in a decrease in condensing pressure, which in turn led to a decrease in compressor operating time, as shown in View A. Comparing the effect of all PCM materials used, an improvement in condenser temperature was evident, despite the lack of direct application to the condenser.

#### Compressor surface temperature

**Fig. 9:**
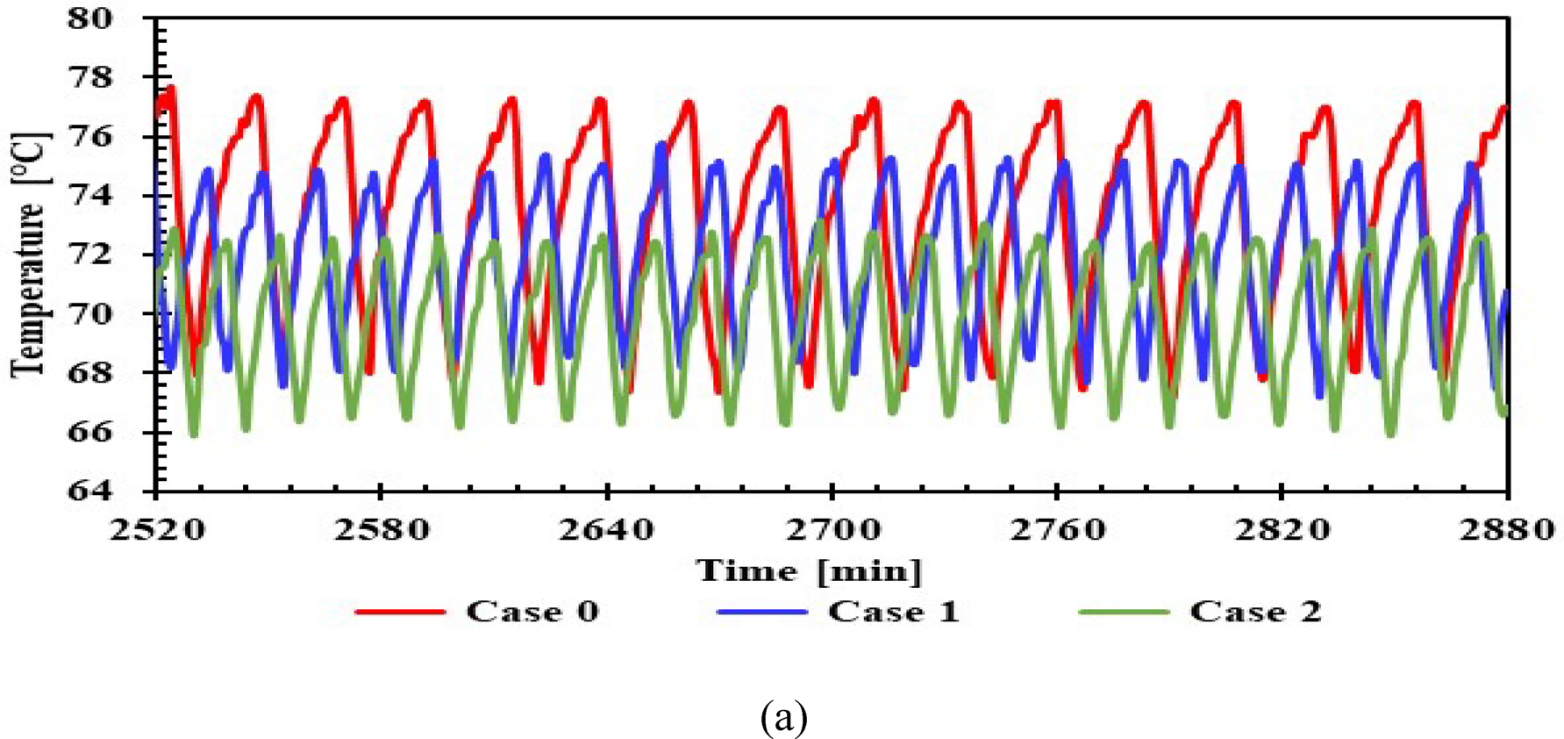

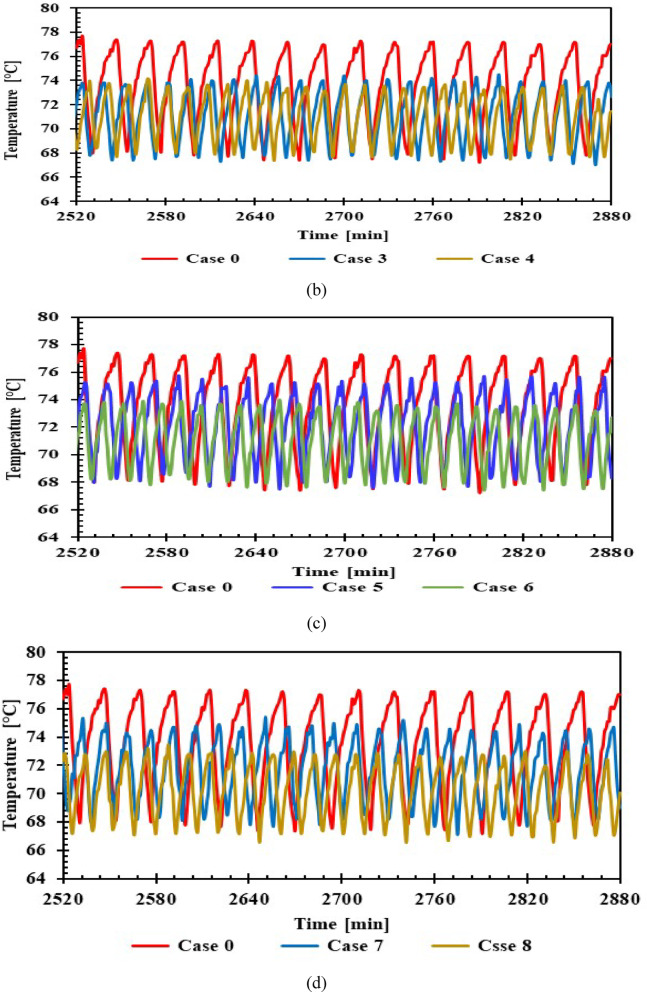
Temperature of compressor surface with and without PCMs over time (**a**,**b**) 1 L, (**c**,**d**) 1.5 L.

Figure 9a–d shows compressor surface temperatures in different scenarios with and without PCM. In case 0, which shows the compressor surface temperature, the temperature peaked at 77.8 °C during the operating cycle. The other cases demonstrate the extent of the improvement in this temperature reduction achieved by PCM. A decrease of approximately 3.1 to 5.5 °C was achieved with a 1 LPCM, whereas the 1.5 L PCM showed a more minor improvement, with a decrease of approximately 2.5 to 4 °C. This is because increasing the PCM size regarding the appropriate size creates a thermal load on the compressor, requiring a longer operating period. However, it was an improvement compared to not having a PCM. During the off cycles, the compressor surface temperature in some cases (4, 5, and 6) was nearly equal to that in the absence of PCM. The maximum reduction of 2 °C was achieved in Case 2. The effect of the PCM on the compressor surface was more pronounced during the operating cycles, due to the shorter cycle time compared to operation without PCM. The analysis showed that refrigerators with PCMs had reduced cycle times, thereby reducing energy consumption, and recorded the highest total number of cycles across all scenarios.

#### Freezer inside temperature

**Fig. 10:**
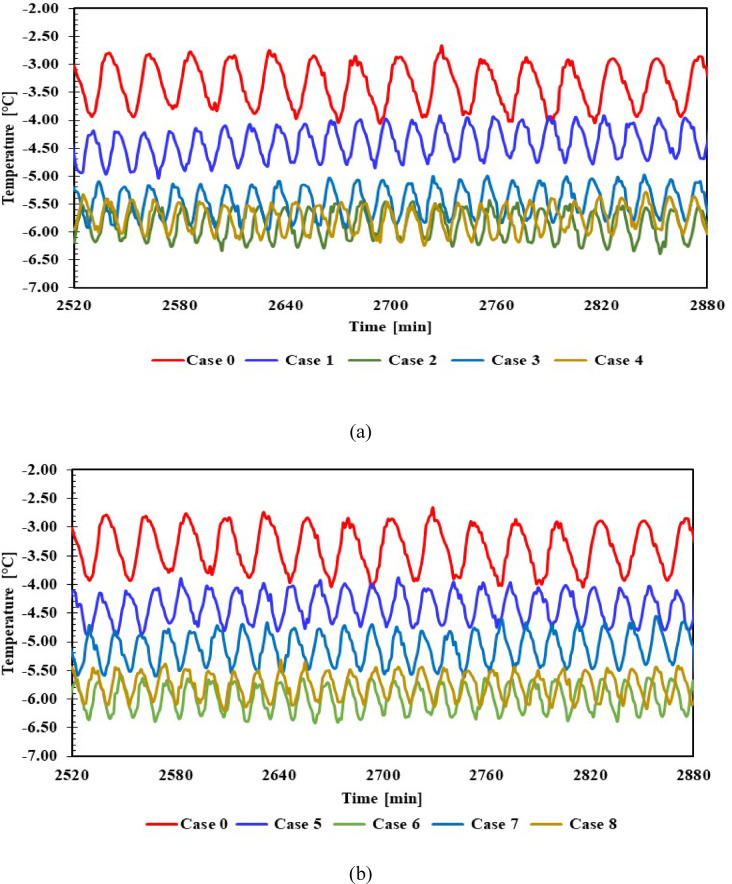
Temperature inside the freezer with and without PCMs over time (**a**) 1 L, (**b**) 1.5 L.

Figure 10 illustrate the internal temperatures of the evaporator with 1 L (cases:1,2,3,4) and 1.5 L (cases:5,6,7,8) of (PCMs) and without PCM (case :0). The temperature variation between maximum and minimum at on/off cycle for cases; 0 (without PCM) between -2.75 °C /-3.8 °C, and with average -3.2 °C, case 1: The temperature was roughly -3.9 °C with 1 L of PCM. Compared to without PCM, KNO_3_ improved performance by lowering the temperature to -6.3 °C. KCl showed similar results across the two sizes, approximately, while water lowered the temperature to -4.9 °C. NaCl reached -5.9 °C with 1 L and -5.5 °C with 1.5 L.

It was found that, across all PCM-equipped refrigerators, the evaporator temperature fluctuated over a greater number of cycles and within a narrower range than for those without PCM. All cases showed a 79.4% improvement in freezer compartment temperature, indicating high stability of the compartment’s internal temperature. This, in turn, raises the COP for each PCM case, with the refrigerating impact increasing and the compressor power decreasing.

#### Refrigerator compartment temperature

**Fig. 11:**
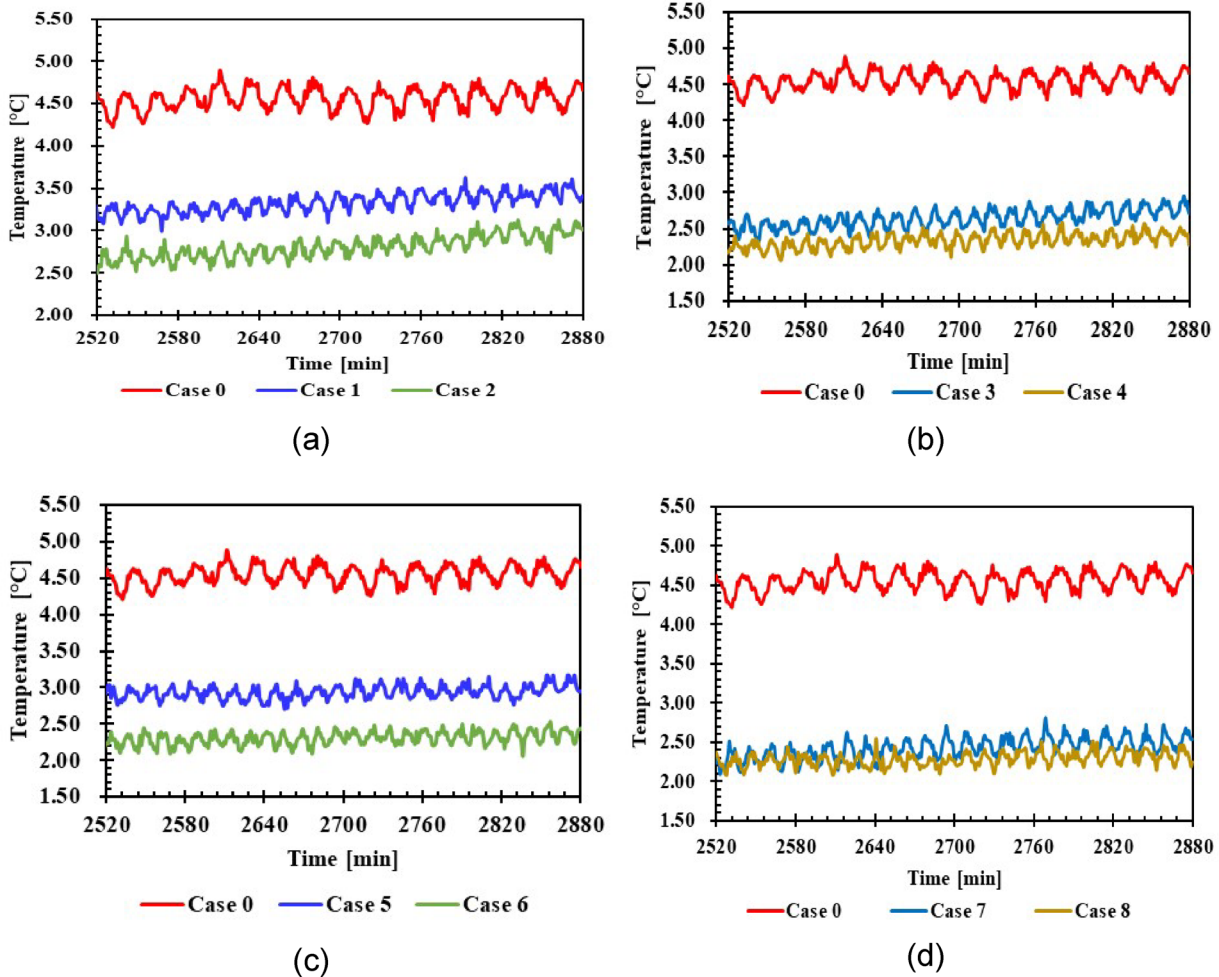
Average temperature refrigerator compartment with and without PCMs over time (**a**,**b**) 1 L, (**c**,**d**) 1.5 L.

Figure 11 illustrates the impact of PCM on the temperatures within the refrigerator compartment for various cases, including 1 L and 1.5 L of PCM, compared to the case without PCM. The temperature in case 0 was 4.3 °C, compared to the PCM case. In cases 1, 2, 3, and 4, the temperature decreased to 3.1 °C, 2.6 °C, 2.4 °C, and 2.2 °C, respectively. In contrast, the temperatures in cases 5, 6, 7, and 8 decreased to 2.8 °C, 2.2 °C, 2.2 °C, and 2.1 °C, respectively. The results of cases 5, 6, and 8 showed more stable temperatures.

The results indicated the extent of PCM’s impact on refrigerator compartment temperatures. Heat absorption by the PCM helps. Heat absorption by the PCM enhances natural convection in the refrigerator compartment. Where shown that the 1.5 L PCM was superior in achieving a temperature improvement of approximately 51% using KNO_3_ solution, in addition to the results of cases 5, 6, and 8 showed more stable temperatures. This is due to the PCM container being located below the evaporator and within the refrigerator compartment, which allows direct contact with the compartment air.

#### Refrigerator vegetable drawer temperature

**Fig. 12:**
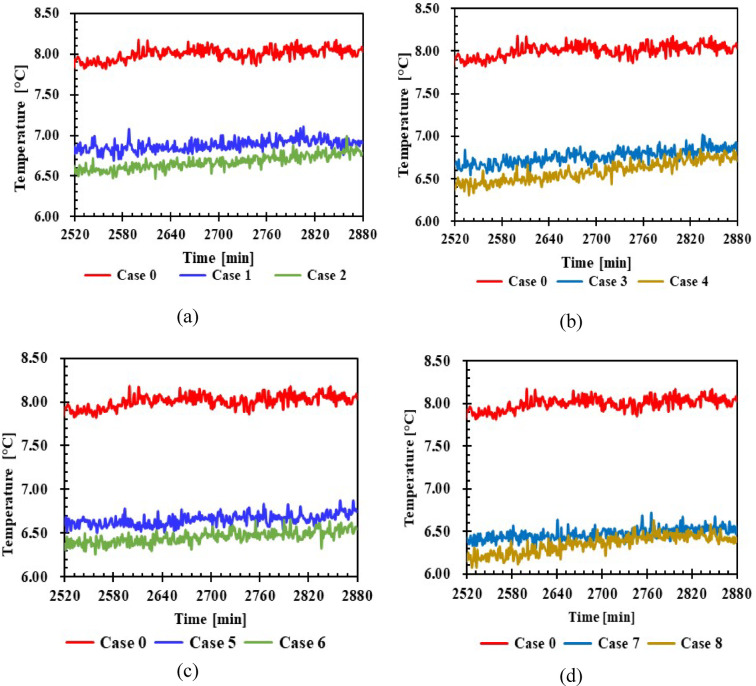
Temperature inside the vegetable drawer with and without PCMs over time (**a**,**b**) 1 L, (**c**,**d**) 1.5 L.

Figure 12 shows that introducing PCM reduced the temperature of the fresh vegetable drawer by 1–1.7 °C compared to when PCM was absent. The results indicate temperature improvements of 19.1% and 22.4% with 1 L and 1.5 L volumes, respectively, due to PCMs generally having the highest heat capacity.

#### Refrigerator door temperature

**Fig. 13:**
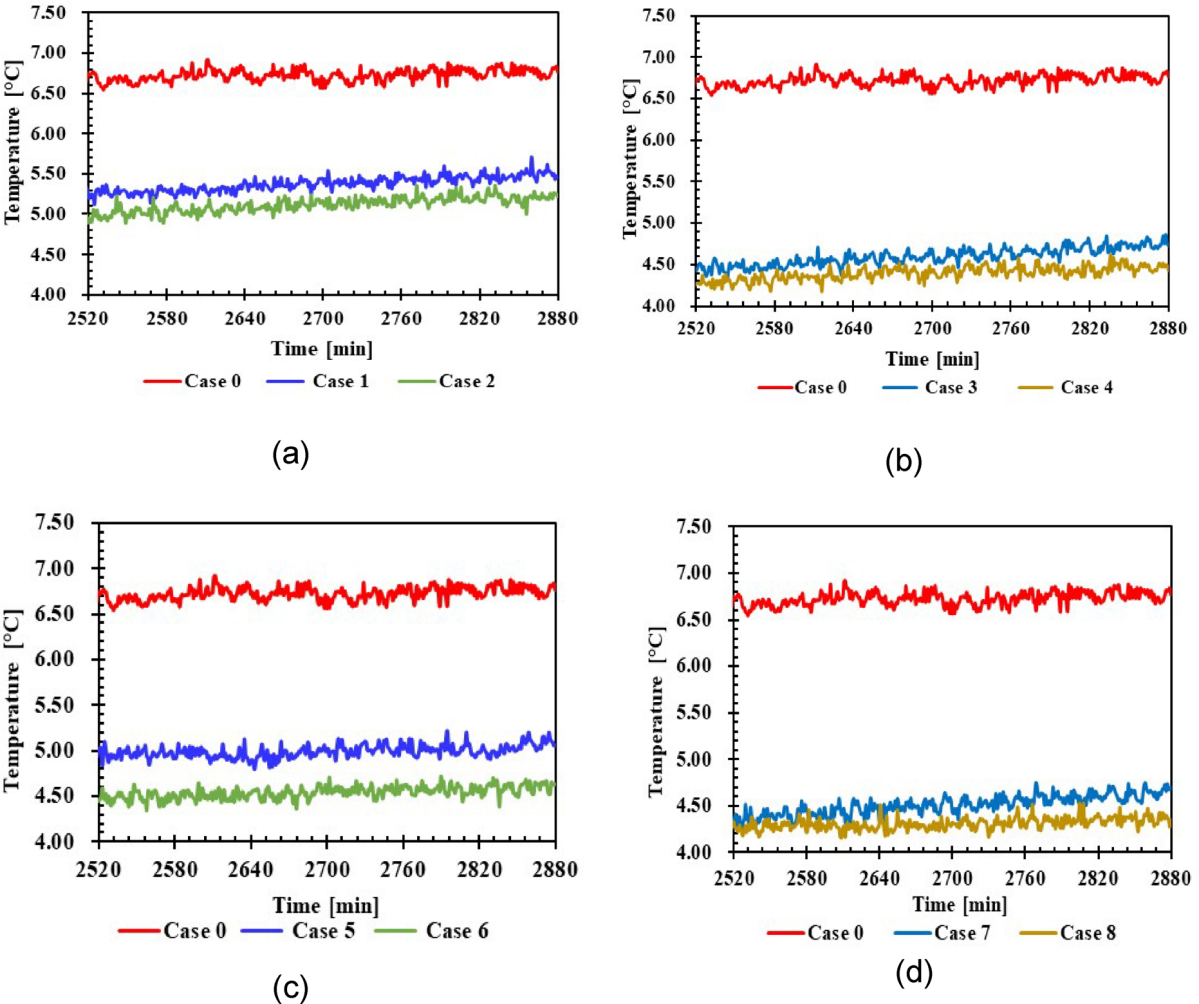
Average temperature door with and without PCMs over time (**a**,**b**) 1 L, (**c**,**d**) 1.5 L.

Figure 13 illustrates the impact of PCM on refrigerator door temperatures for various cases, including 1 L and 1.5 L of PCM, compared to the case without PCM. The variations observed in cases 3, 4, 7, and 8 are more pronounced than those in cases 1, 2, and 5. Notably, there is convergence in the temperature differences among cases 1, 2, 3, 4, 7, and 8, in contrast to the discrepancies observed in cases 5 and 6, particularly compared to case 0, as depicted in Fig. 13. The average temperature difference in cases 3, 4, 7, and 8 is recorded at 4.25 °C, whereas in cases 1, 2, 5, and 6, the average temperature is noted to be 4.75 °C.

### Impact of PCMs on refrigeration temperature during a power outage

The temperature distribution in the freezer and refrigerator was assessed during a 60-min off-duty period after 48 h of operation, with and without PCM. The aim was to evaluate the effect of PCM on the refrigerator’s internal temperatures during power outages. The evaluation was conducted under the same ambient conditions specified in the previous experiments.

#### Freezer inside temperature

**Fig. 14:**
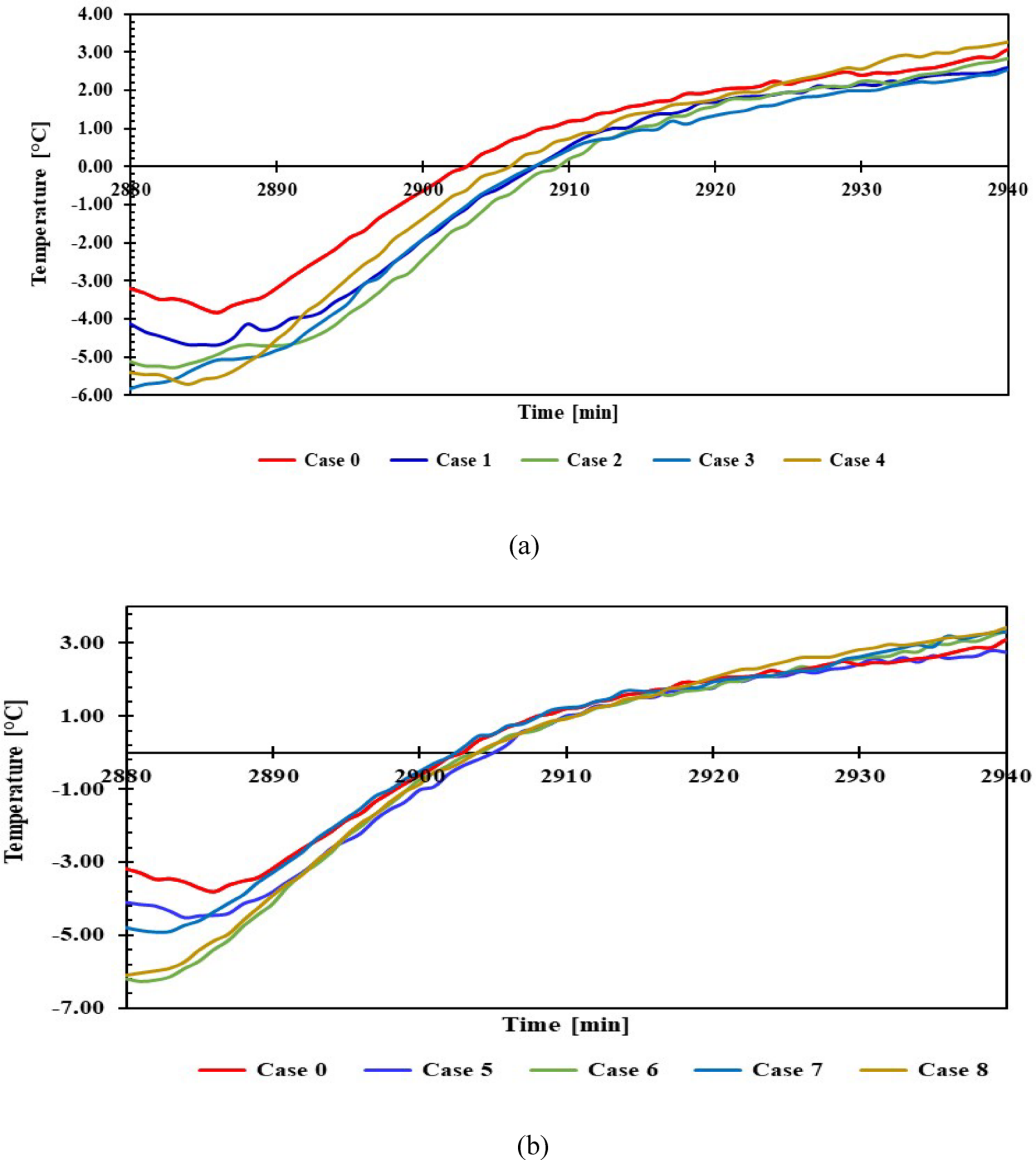
Temperature of the freezer with and without PCM during a power outage (**a**) 1 L, (**b**) 1.5 L.

Figure 14 shows the temperatures inside the freezer compartment when power was disconnected, for 1 L and 1.5 L of PCM, compared to without PCM. The results indicated that using 1 L of PCM maintained the freezer compartment temperature below 0 °C for approximately 30 min. PCM continued to improve the temperature over the course of an hour, but after 50 min, the KCl solution showed a higher compartment temperature than without PCM. Furthermore, the continued maintenance of the compartment temperature was superior to the 1.5 L size. Use 1.5 L, and after approximately 50 min, the compartment temperature rose higher than it did without the PCM, which indicates that the materials, after 50 min, represent a thermal load. In all cases with and without PCM, the temperature consistently rises until it nears the midpoint, after which the rate of increase slightly declines.

#### Refrigerator compartment temperature

**Fig. 15:**
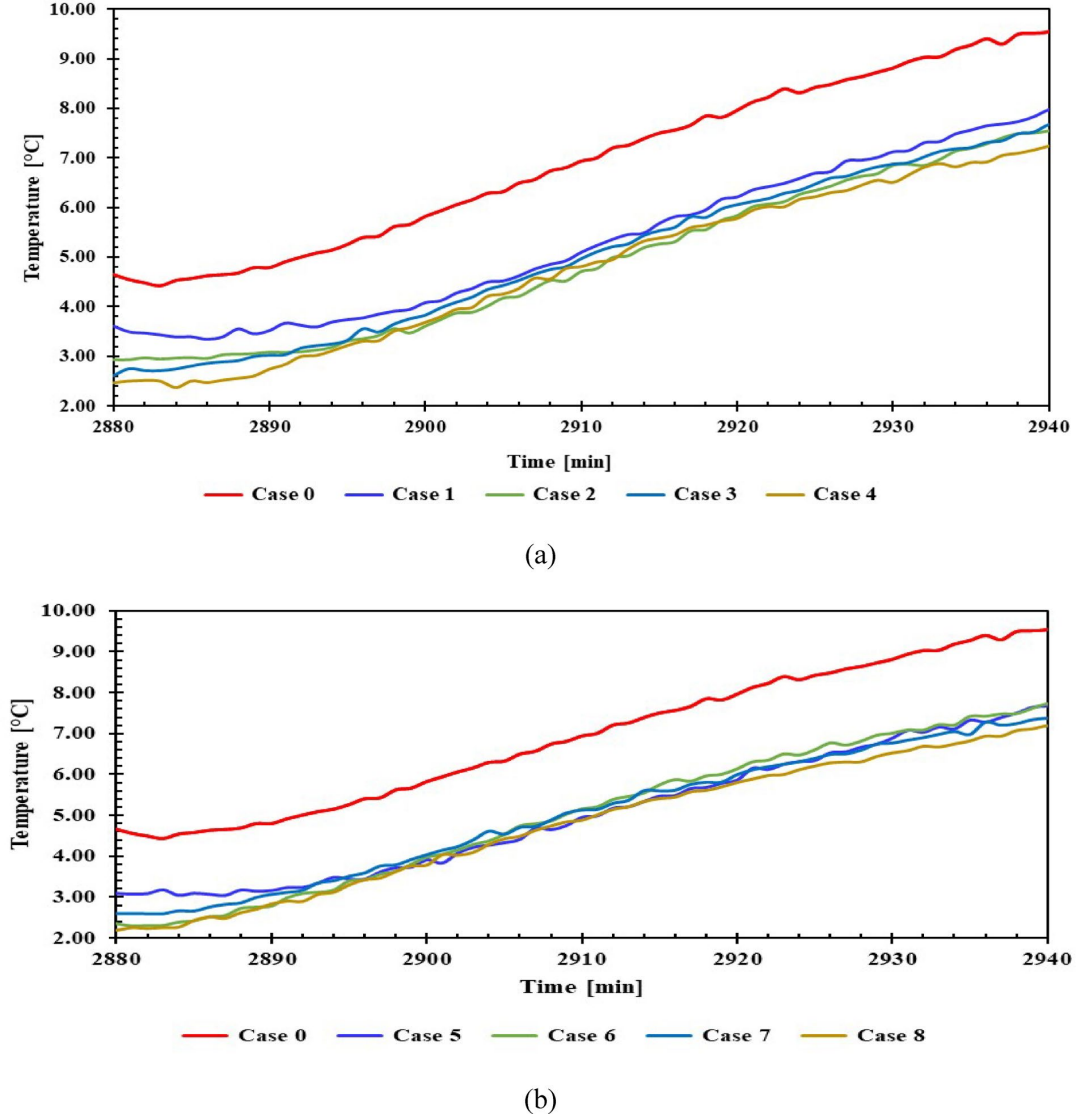
Temperature inside the compartment with and without PCM during a power outage (**a**) 1 L, (**b**) 1.5 L.

Figure 15 shows the temperatures inside the refrigerator compartment when power was disconnected, for 1 L and 1.5 L of PCM, compared to without PCM. It is observed that cases 3, 4, 7, and 8 reach 7.2 °C after 60 min, whereas cases 1 and 2 reach 7.8 °C, and cases 5 and 6 reach 7.5 °C. In contrast, case 0 records a temperature of 9.8 °C. All results indicate a positive effect of PCM materials on the compartment, maintaining a 2 °C lower temperature within 60 min compared to without PCM.

### Energy consumption

**Fig. 16:**
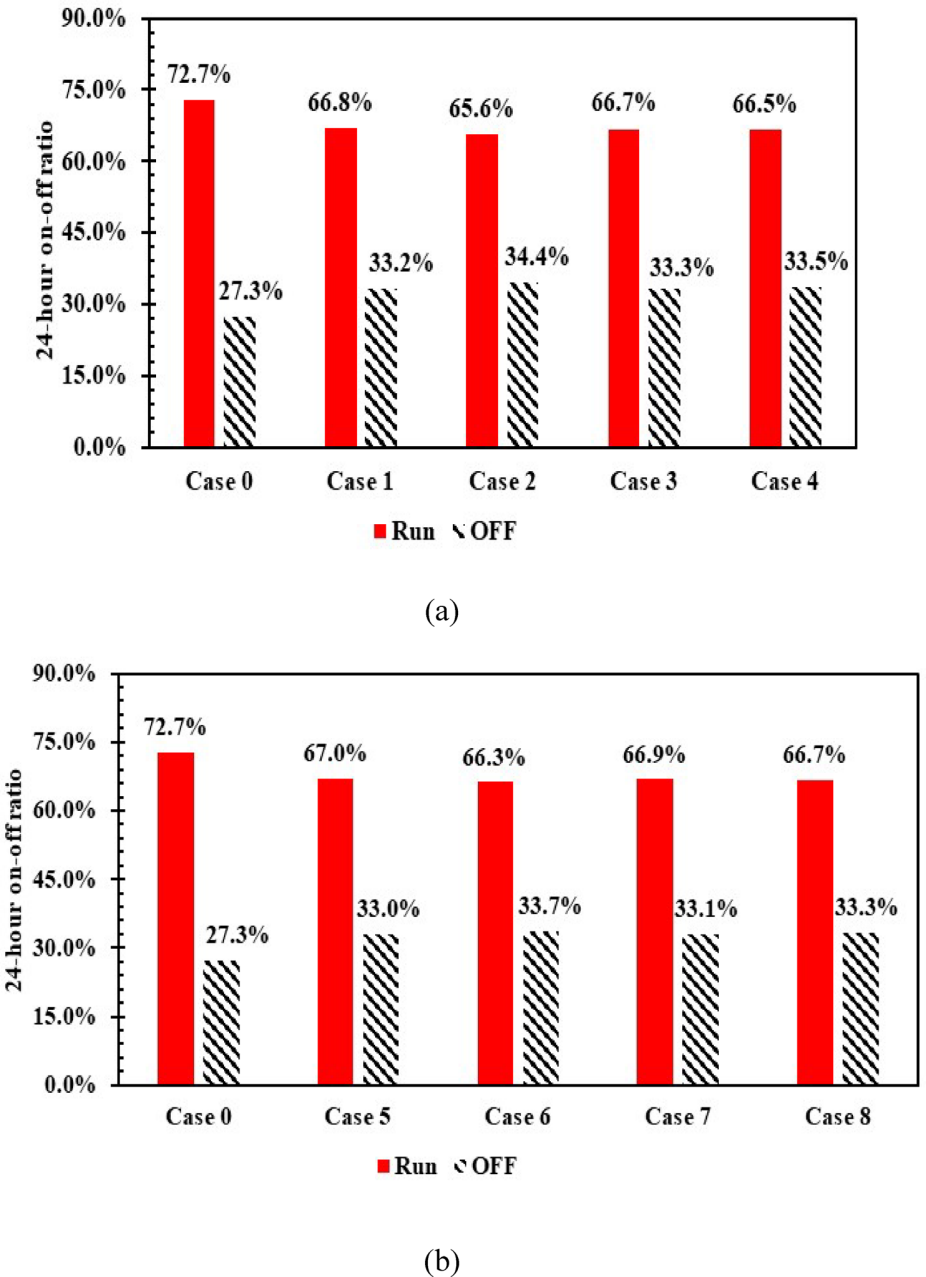
Run and off time with and without PCM over 24 h (**a**) 1 L, (**b**) 1.5 L.

The application of PCM demonstrated a significant impact on reducing compressor operation periods over 24 h, indicating that part of the heat load is removed by the PCM, thereby reducing compressor operating times. (see Fig. 16). When utilizing 1 L and 1.5 L of PCM, the compressor operating periods decreased by 7.1% in case 2. In contrast, the lowest percentage of decrease was achieved in case 5, which amounted to 5.7%. In contrast to the absence of PCM.

**Fig. 17:**
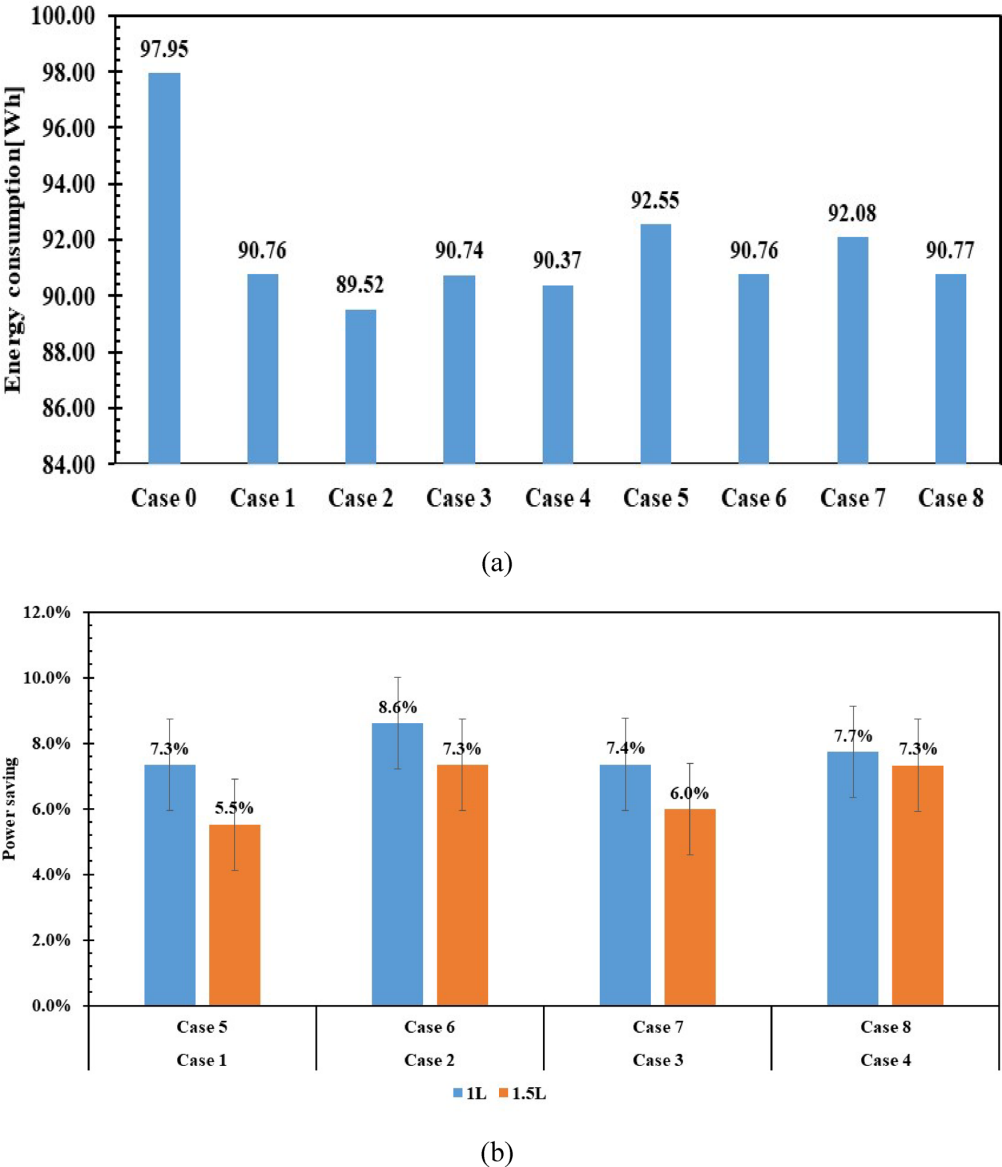
Energy consumption in a refrigerator with and without PCMs (**a**) Power saving (**b**).

Figure 17 illustrates energy consumption and savings for 1 L and 1.5 L of PCM compared to the case without PCM. Figure 17a shows that the energy consumption for case 0 is 97 Wh, the highest among all cases. The effect of PCM on reducing energy consumption is also evident in reduced compressor operating time, as shown in Fig. 16. Energy savings reached 8.6% in case 2, compared to 7.3% in case 6. Case 5 has the lowest energy-saving percentage of 5.5%. By comparison to the improvement in energy consumption in cases 1 and 5, and the improvement discussed by Rahimi et al.^40^ In their research article, using 2.3928 kg of H_2_O, the results indicate that increasing the PCM size reduces the improvement in electrical consumption. The results indicate that increasing the PCM mass does not necessarily improve energy consumption. Due to several factors, including heat transfer and thermal resistance within the PCM layers, which reduce the PCM’s effective utilization. underscores the importance of determining the optimal mass and thickness of the PCM relative to the PCM’s position and the design container. It is also crucial to consider whether heat transfer within the refrigerator relies on natural or forced convection. Neglecting any of these factors increases the compressor’s load, resulting in higher energy consumption. The KCl solution outperformed the NaCl solution in reducing electricity consumption at both volumes, achieving 7.7% and 7.3% savings using 1 L and 1.5 L, respectively, while the NaCl solution achieved 7.4% and 6% savings.

### Effect of PCMs on (COP)

**Fig. 18:**
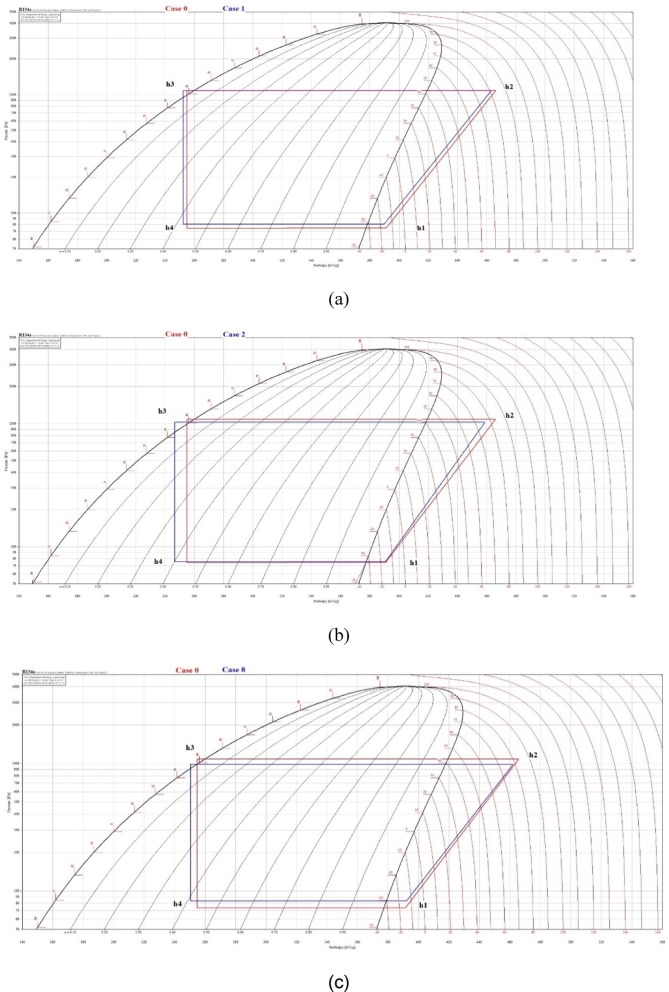
P–h cycles of the refrigerator with and without PCM R134a, (**a**) case 1, (**b**) case 2, (**c**) case 8.

The impact of PCM use on refrigerator performance is demonstrated in Fig. 18, which shows P–h cycles for cases 0, 1, 2, and 8, with and without PCM. The cooling effect and compressor power exhibited slight enhancements in case 1 of the blue cycle (H_2_O) when compared to case 0 of the red cycle (without PCM), as illustrated in Fig. 18a. In case 2, there was a significant increase in the cooling effect, accompanied by a reduction in condensing pressure and a decrease in compressor work. Consequently, this is evident in the (COP), as illustrated in Fig. 18b.

**Fig. 19:**
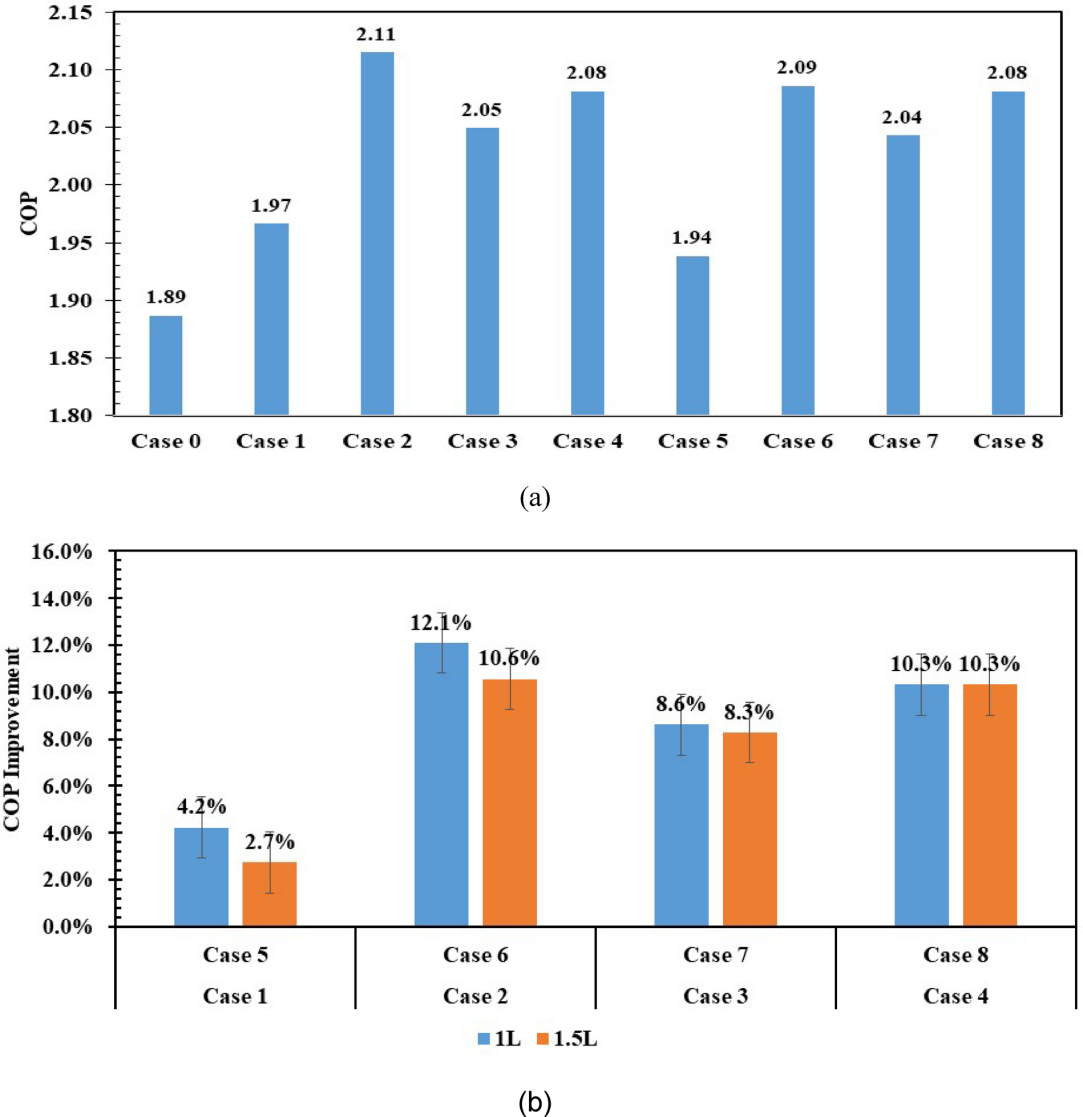
COP in different cases, refrigerator with and without PCMs (**a**) improvement in COP (**b**).

The COP of the refrigerator is evaluated with 1 L and 1.5 L of PCM, compared to that without PCM. The COP is derived from the pressure-enthalpy (P–h) diagram for refrigerant R-134a using the following equation, as in Fig. 19a,b.

$$COP=\frac{h1-h4}{h2-h1}$$

The highest COP is for case 2 (Eutectic1 with 1 L), followed by the other cases, with an improvement of 12.1% over without PCM, as shown in Fig. 19b.

Figure 19b compares the percentage improvement in COP resulting from PCM use for the 1 L and 1.5 L cases. Indicates that the improvement percentages reached 12.1% and 10.6% with KNO_3_ solutions at volumes of 1 L and 1.5 L, respectively. Meanwhile, the KCl solution achieved 10.3% in both cases. The (10% NaCl + 90% H_2_O) mixture ranked third in terms of COP improvement, with 8.6% and 8.3% for 1 L and 1.5 L volumes, respectively. Meanwhile, H_2_O achieved an improvement percentage of 4.2% and 2.7% at volumes of 1 L and 1.5 L, respectively.

## Limitations

This study has certain limitations related to the experimental evaluation of phase change materials and some advanced thermal. The absence of differential thermal scanning tests is a limitation of this study. The aim is to investigate the system’s performance in the presence of these materials. Describing the thermal properties of materials requires precise materials science testing. This highlights the need for future studies to investigate their stability and thermal behavior

## Conclusions

An experimental study was conducted to evaluate the integration of phase change materials into home refrigerators. KNO_3_, KCl, and NaCl were mixed at a ratio of 10% with 90% H_2_O, and H_2_O was used at a rate of 100%. The mixture was used at volumes of 1 L and 1.5 L in a stainless-steel container placed under the evaporator, ensuring contact with the evaporator coils. The experiments were conducted in a 289-L, single-door refrigerator operating on R-134a refrigerant inside an insulated room, where the ambient temperature was maintained at 32 °C and the humidity at 55%. The experiments were conducted under eight different scenarios with PCMs; a comparison was made between the PCMs and the scenario without PCMs. The study aimed to conduct a comprehensive analysis and comparison of the effect of these materials on refrigerator parameters (COP, energy consumption, temperatures within the cooling and freezing compartments, and compressor and condenser surface temperatures). The following conclusions were drawn:

- The implementation of PCM contributed to a reduction in temperatures within the refrigerator, with the internal compartment temperatures decreasing by 1 °C to 3 °C. Furthermore, all materials demonstrated PCM’s capacity to mitigate temperature variations in the refrigerator environment.
- The temperature of the condenser and the compressor surface decreased by approximately 1.6 °C and 6.3 °C, respectively.
- (10% KNO_3_ + 90% H_2_O) wt achieved a 7.1% increase in compressor shutdown time using a 1 L volume, compared to the same mixture using a 1.5 L volume, which achieved a 6.4% increase.
- Improving the COP of the refrigerator by 12.1% using 1 L of (10% KNO_3_ + 90% H_2_O) wt. The mixture was compared to the same mixture for a volume of 1.5 L, which showed an improvement of 10.6%. At the same time, the (10% KCl + 90% H_2_O) mixture achieved a 10.3% increase in COP at both volumes.
-The (10% NaCl + 90% H_2_O) mixture ranked third in terms of improvement in COP, with 8.6% and 8.3% for the 1 L and 1.5 L volumes, respectively.
- Energy consumption using KNO_3_ decreased by 8.6% for a 1 L volume and 7.3% for a 1.5 L volume. The lowest improvement in energy consumption was achieved using 100% H_2_O, resulting in a 5.5% reduction for a 1.5 L volume.
- Using the 1 L size had a positive effect on energy consumption and COP, while using the 1.5-L size showed a more effective effect on cooling compartment temperatures.

The results confirm that the use of PCMs is an ideal solution for energy conservation and achieving stable temperatures, sustainable development, reducing operating times, and increasing COP.

## Data Availability

All data generated or analysed during this study are included in this published article.

## List of symbols

h_1_: Enthalpy at the inlet to the compressor (kJ/kg)
h_2_: Enthalpy at the exit compressor (kJ/kg)
h_3_: Enthalpy at the inlet to the compressor (kJ/kg)
h_4_: Enthalpy at the exit from the expansion valve (kJ/kg)
P_H_: Pressure at the compressor outlet (kPa)
P_L_: Pressure at the compressor inlet (kPa)
T_1_, T_2_, T_3_: Temperatures inside the freezer (°C)
T_4_, T_5_, T_6_: Temperatures inside the compartment (°C)
T_7_: Temperature inside the Vegetable drawer (°C)
T_8_, T_9_: Temperatures at the door (°C)
T_out_: Temperature at the compressor outlet (°C)
T_in_: Temperature at the compressor inlet (°C)
T_H_: Condenser top temperature (°C)
T_Mid_: Condenser midpoint temperature (°C)
T_L_: Condenser bottom temperature (°C)
T_F_: Condenser fin temperature (°C)
T_PCM_: Phase change material temperature (°C)
T_S_: Container surface temperature (°C)

## Abbreviations

COP: Coefficient of performance
PCM: Phase change material
VCR: Vapor compression refrigeration
CFD: Computational fluid dynamics
